# Effect of *Cocos nucifera oil* on tensile strength of two different tissue conditioners at different time intervals

**DOI:** 10.6026/9732063002001191

**Published:** 2024-09-30

**Authors:** Pushkar Gupta, Abhishek Pathak, Sneha S Mantri, Harsh Chansoria, Shivakshi Chansoria, Swati Solanki

**Affiliations:** 1 Department of Prosthodontics and Crown & Bridge, Hitkarini Dental College and Hospital, Jabalpur, M.P., India; 2 Department of Prosthodontics and Crown & Bridge, Government College of Dentistry, Indore, M.P., India; 3 Department of Oral Medicine and Radiology, Government College of Dentistry, Indore, M.P., India

**Keywords:** *Candida albicans*, *Cocos nucifera oil*, tensile strength, tissue conditioners, soft liners

## Abstract

Tissue conditioners are a short-term soft liner that is frequently used in dentistry to improve the condition of abused denture-bearing tissues, particularly in patients with denture stomatitis. Topical application of antifungal agent has multiple disadvantages which can be negated by incorporation of an herbal agent like *Cocos nucifera oil* into the tissue conditioner, which can enhance its mechanical properties like tensile strength, while aiding in reduction of *C.albicans* count in the oral cavity. Therefore, it is of interest to evaluate the tensile strength of Visco-gel and GC soft liner with and without 10% w/w *Cocos nucifera oil* on different time intervals. 32 dumb-belled shaped samples of each Visco-gel and GC soft liner were divided into two groups of 16 each (with and without 10% w/w Cocos nucifera oil). All the samples were stored in sterile glass jars with distilled water at 37°C. 4 samples from each group were tested for tensile strength on day 1, day 3, day 7 and day 14. Tensile strength test was performed on a Universal testing machine with computer control, data acquisition, and data analysis software. Incorporation of 10% w/w *Cocos nucifera oil* statistically increases the tensile strength of Visco-gel and GC soft liner, from day 1 to day 14, after immersion in distilled water.

## Background:

Tissue conditioners are a short-term soft liner that is frequently used in dentistry [1]. Tissue conditioners are also effective in patients for making functional impression as described by Winkler [2] for temporary relining of ill-fitting denture and immediate denture and during implant healing [3, 4]. The most common opportunistic infection among removable denture wearers is denture stomatitis. Despite having a recognized multimodal etiology, 93% of cases of denture stomatitis are caused by candida infection, specifically by *Candida albicans* [5, 6]. Eliminating local irritants, addressing the damaged tissue, nutrition counselling, changing the out-dated prosthesis, systemic examination, appropriate occlusal scheme and correcting any pre-maturities are all part of an effective treatment strategy for denture stomatitis [6]. Antifungals applied topically or taken systemically can effectively reduce the signs and systems of dentures stomatitis [6, 7]. Antifungal medicines should not be applied topically, since saliva might wash the medication away and leave an inadequate concentration at the site of action [7, 8-9]. Large dosages of medications carrying a high risk of side effects are necessary for systemic delivery [8]. Soft liners now include antifungal ingredients to help with these drawbacks. Addition of any substance into a tissue conditioner can alter its mechanical properties such as tensile strength. Tensile strength refers to the maximum material strength under tension, being considered a fundamental attribute for rubber materials [10, 11]. The tensile properties are significant in the overall examination of performance and quality of these materials. Since herbal medications are readily available, have few or no adverse effects, and are more affordable than other organic and inorganic chemicals, hence natural and herbal medications are preferred. *Cocos nucifera* or coconut oil is one such natural oil that possesses antifungal and antibacterial properties [12]. In this study, 10% w/w *Cocos nucifera oil* was chosen for its substantial antifungal activity against *C. albicans*. Therefore, it is of interest to evaluate the tensile strength of Visco-gel and GC soft liner with and without 10% *Cocos nucifera oil* on different time intervals.

## Materials and Methods:

**Figure 1:**
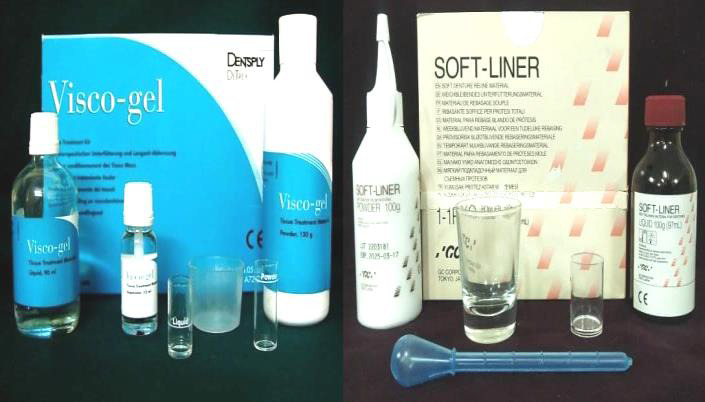
Visco-gel and GC soft liner

This *in vitro* study was done in the department of Prosthodontics. In this study two commonly used tissue conditioners, Visco-gel and GC soft liner (Figure 1) were used with and without 10% w/w *Cocos nucifera oil*, at different time interval for evaluation of tensile strength.

## Preparation of moulds:

Using modelling wax, dumb belled-shaped wax samples with a cross-sectional area of 33 x 6 x 3 mm were created in accordance with ASTM D412 guidelines [13]. These wax samples were de-waxed to create moulds after being invested in the lower portion of the flask using a type IV die stone [12].

## Preparation of samples:

16 samples of Visco-gel (C1) and GC soft liner (C2) without 10% w/w *Cocos nucifera oil* and 16 samples of Visco-gel (T1) and GC soft liner (T2) with 10% w/w *Cocos nucifera oil* were prepared. (T1) samples were prepared by mixing 3g (one measure) of powder with 2.2ml (one measure) of liquid for Visco-gel and (T2) samples were prepared by mixing 2.2g (one measure) of powder with 1.8g (one measure) of liquid according to the manufacturer's instructions for 30 seconds. Samples of group (T1) and (T2) were prepared by adding 1 ml of 10% w/w *Cocos nucifera oil* per sample. Both components were evenly handled, later put into the mould's lower part, and given time to gel. After retrieving the set samples, excess was cut with a Bard Parker knife.

## Grouping of the samples:

**Figure 2:**
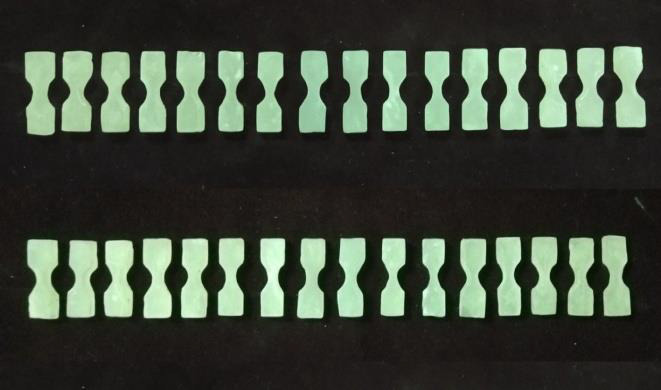
Samples of Visco-gel

**Figure 3:**
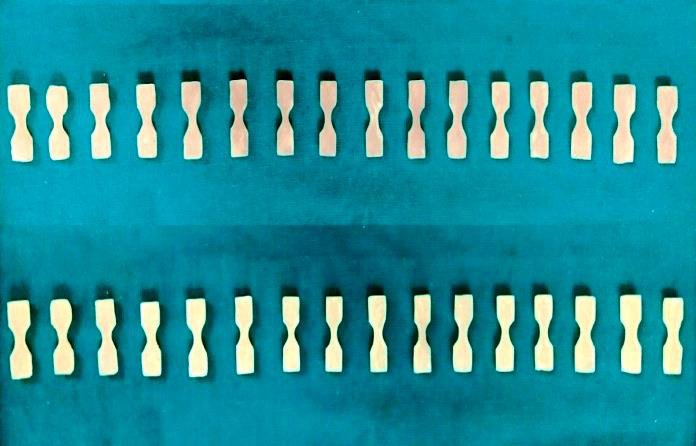
Samples of GC soft liner

32 dumbbell shaped samples of each Visco-gel (Figure 2) and GC soft liner (Figure 3) were divided into two groups of 16 each. Four samples from each group were tested for tensile strength on first day, third day, seventh day and fourteenth day.

## Storage of samples:

Before the tensile strength testing, all of the samples were kept for 24 hours, 3 days, 7 days, or 14 days in sterile glass jars with distilled water at 37°C [13, 14]. Every 24 hours, the distilled water was replaced.

## Measurement of tensile strength:

**Figure 4:**
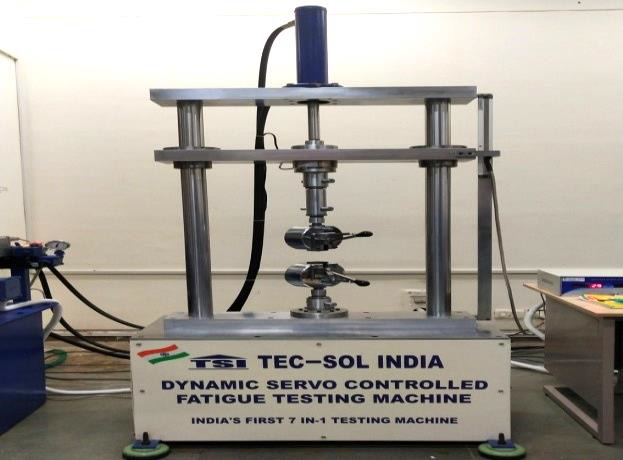
Universal testing machine

A Universal Testing Machine (TEC-SOL INDIA) (Figure 4) with computer control, data gathering and data analysis software (Tec-sol software v2.18.713, Tec-Sol-India, Corp.) was used to measure the tensile strength at a cross-head speed of 40 mm/min [15]. In order to expose only the center region of the specimens during testing, a claw made especially for this test was developed [14]. The values for the tensile strength were found in Newton (N).

## Results:

The obtained data was analyzed from 64 samples of which, 32 were of Visco-gel (VG) and 32 ofGC soft liners (SL). The data was analysed using one independent sample t-test (unpaired test) for intergroup comparison. ANOVA test was used to compare the intragroup comparison at different time intervals in individual groups. Post hoc turkey test was done to compare mean differences between the intragroup comparisons at different time intervals. The p value < 0.05 was considered statistically significant. Data analyses were performed using version 26.0 of the Statistical Package for Social Sciences (IBM Corporation, Armonk, New York, USA).

Table 1 Depicts the mean tensile strength values between control group C1 (Visco-gel) and control group C2 (GC soft liner) without 10% w/w *Cocos nucifera oil*. The mean tensile strength value of Visco-gel at day 1 was 1.35±0.05 and that of GC soft liner at day 1 was 1.20±0.80. The mean tensile strength value of Visco-gel at day 3 was 2.22±0.09 and that of GC soft liner at day 3 was 2.07±0.09. The mean tensile strength value of Visco-gel at day 7 was 3.25±0.19 and that of GC soft liner at day 7 was 2.97±0.12. The mean tensile strength value of Visco-gel at day 14 was 3.22±0.12 and that of GC soft liner at day 14 was 3.05±0.05.Table 2 Depicts the mean tensile strength values between test group T1(Visco-gel) and test group T2 (GC soft liner) with 10% w/w *Cocos nucifera oil*. The mean tensile strength value of Visco-gel at day 1 was 1.45±0.26 and that of GC soft liner at day 1 was 1.42±0.17. The mean tensile strength value of Visco-gel at day 3 was 2.4±0.21 and that of GC soft liner at day 3 was 2.15±0.012. The mean tensile strength value of Visco-gel at day 7 was 3.35±0.12 and that of GC soft liner at day 7 was 3.15±0.05. The mean tensile strength value of Visco-gel at day 14 was 3.55±0.12 and that of GC soft liner at day 14 was 3.32±0.15.

## Discussion:

The use of *Cocos nucifera oil* in various tissue conditioners to treat denture stomatitis has not been extensively studied. Thus, *Cocos nucifera oil* and two distinct tissue conditioner types (Visco-gel and GC soft liner) were employed in the current investigation. Tissue conditioners reportedly last for 14 days, according to Graham *et al.* findings [15, 16] and it is indicated that they remain effective over this time. The mean tensile strength value of Visco-gel without 10% w/w *Cocos nucifera oil* increased from 1.35 ± 0.05 to 3.22 ± 0.12 from day 1 to day 14 and GC soft liner increased from 1.20 ± 0.80 to 3.05 ± 0.05 (Table 1). The increased values of mean tensile strength of Visco-gel could be due to the strength of filler-polymer bonding. It was suggested that stronger the filler-polymer bonding, the better would be tensile strength [10, 11]. Additionally, the water is able to absorb the ethanol and ester plasticizers due to the wet environment and the polymeric phase of the gel absorbs this water [17]. The materials gradually harden as a result of the loss of plasticizers. Superior tensile strength of Visco-gel was compared to GC soft liner may potentially be attributed to higher degree of cross-linking and higher strength of the filler-polymer bonding. The mean tensile strength value of Visco-gel with 10% w/w *Cocos nucifera oil* increased from 1.45 ± 0.26 to 3.55 ± 0.12 from day 1 to day 14 and GC soft liner increased from 1.42 ± 0.17 to 3.32 ± 0.15 (Table 2). The mean value of tensile strength of Visco-gel was higher than that of GC soft liner from day 1 to day 14. Given that the tissue conditioner contains 10% w/w of *Cocos nucifera oil*, it is likely that this oil has an impact on the tissue conditioner's structure. The ethanol is absorbed by the polymer particles during the first mixing of the powder and liquid of the substance, which causes the powder particles to swell. Disentanglements between polymer chains then happen. These disentanglements allow the higher molecules of the plasticizers to penetrate between polymer chains. When the polymer chains in the ethanol and plasticizer are homogeneous, gel formation eventually appears [18]. Tensile strength was enhanced by adding 10% w/w *Cocos nucifera oil*; this could have been caused by the tissue conditioner fully gelling. Stronger cohesion between the polymer chains may have resulted from enhanced disentanglement of the polymer beads caused by an increased plasticizer concentration.

Visco-gel and GC soft liner tissue conditioner's differing tensile strength ratings may be the result of a variation in the molecular weight of the polymer; a polymer with a greater molecular weight will dissolve and diffuse more slowly [17]. Visco-gel was shown to have greater tensile strength values than GC soft liner because its molecular weight (179000) is lower than that of GC soft liner (234000) [18,19] and it can be more readily absorbed by plasticizers.

## Conclusion:

Data shows that incorporation of 10% w/w *Cocos nucifera oil* increased the tensile strength of Visco-gel and GC soft liner, from day 1 to day 14, after immersion in distilled water. The tensile strength of Visco-gel with and without 10% *Cocos nucifera oil* was higher than that of GC soft liner.

## Figures and Tables

**Table 1:** Comparison of mean values between control group C1 (Visco-gel) and control group C2 (GC soft liner) without 10% w/w *Cocos nucifera oil*.

**Time interval**	**Group**	**Mean**	**std**	**t-value**	**p-value**
Day 1	Visco-gel	1.35	0.05	2.976	0.06
	GC soft liner	1.2	0.8		
Day 3	Visco-gel	2.22	0.09	2.216	0.06
	GC soft liner	2.07	0.09		
Day 7	Visco-gel	3.25	0.19	2.347	0.06
	GC soft liner	2.97	0.12		
Day 14	Visco-gel	3.22	0.12	2.528	0.06
	GC soft liner	3.05	0.05		

**Table 2:** Comparison of mean values between test group T1 (Visco-gel) and test group T2 (GC soft liner) with 10% w/w *Cocos nucifera oil*.

**Time interval**	**Group**	**Mean**	**std**	**t-value**	**p-value**
Day 1	Visco-gel	1.45	0.26	0.159	0.08
	GC soft liner	1.42	0.17		
Day 3	Visco-gel	2.4	0.21	1.987	0.105
	GC soft liner	2.15	0.12		
Day 7	Visco-gel	3.35	0.12	2.828	0.09
	GC soft liner	3.15	0.05		
Day 14	Visco-gel	3.55	0.12	2.274	0.003
	GC soft liner	3.32	0.15		
